# Supplementary material for: Single cell profiling of γδ hepatosplenic T-cell lymphoma unravels tumor cell heterogeneity associated with disease progression
Source: Cell Oncol (Dordr). 2022 Nov 22;46(1):211–26. doi: 10.1007/s13402-022-00745-x (PMC9947078; doi:10.1007/s13402-022-00745-x)

Figure S1. CT scans of lymph node indicate lymphadenopathy is absent. (A) Red marks left inguinal lymph node (B) Red marks left upper cervical lymph node

A

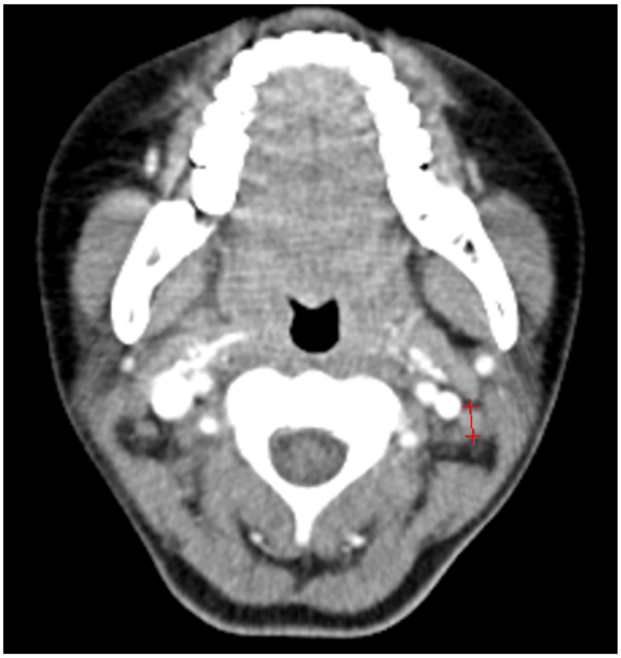

B

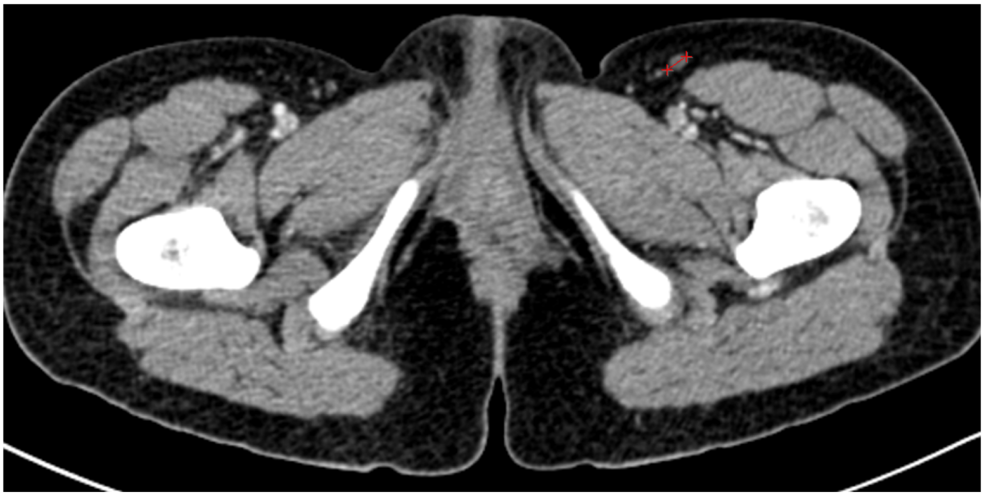

Figure S2. UMAP projections of each sequenced sample.

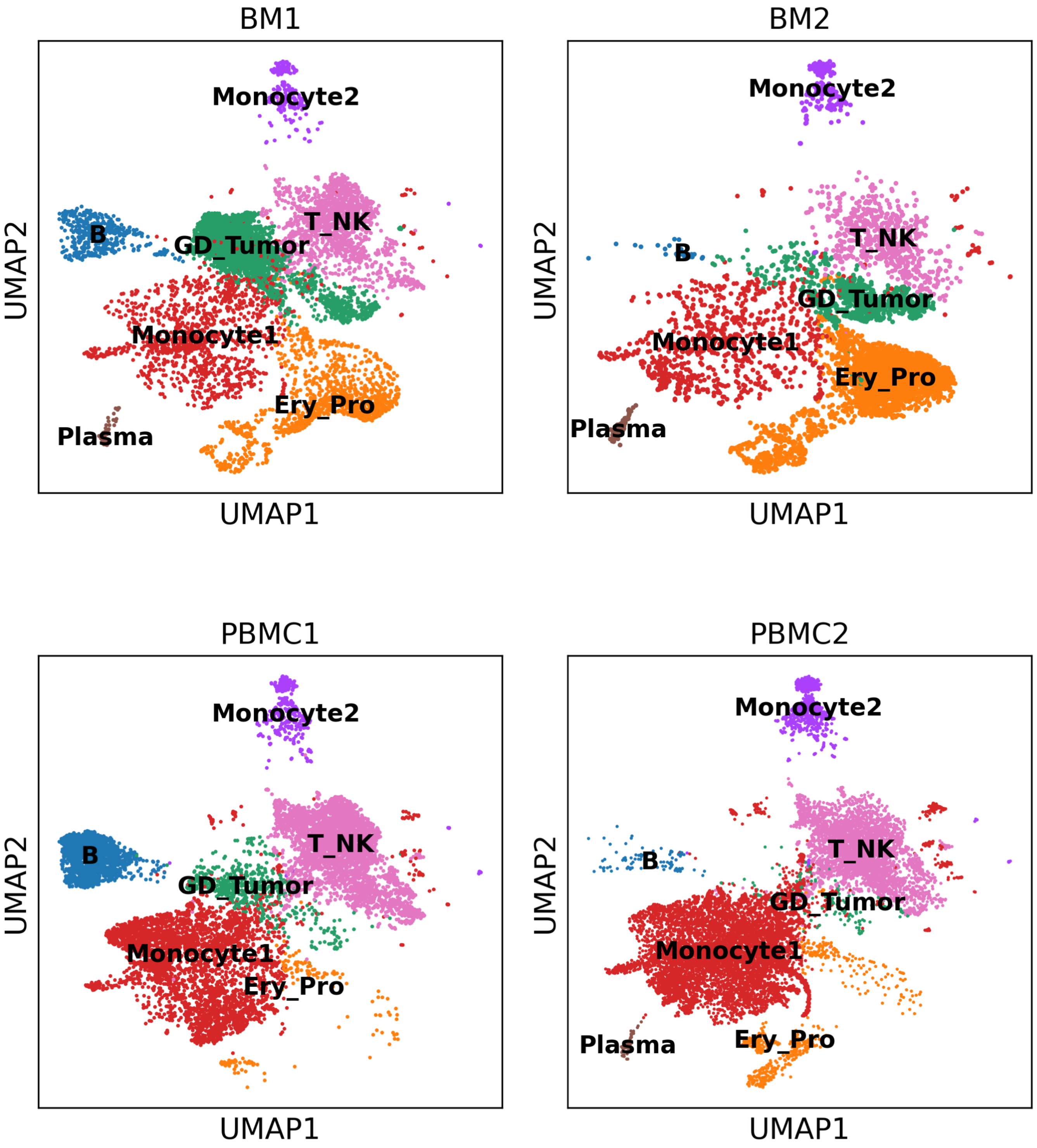

Figure S3. Expression of common markers used for HSTCL diagnosis

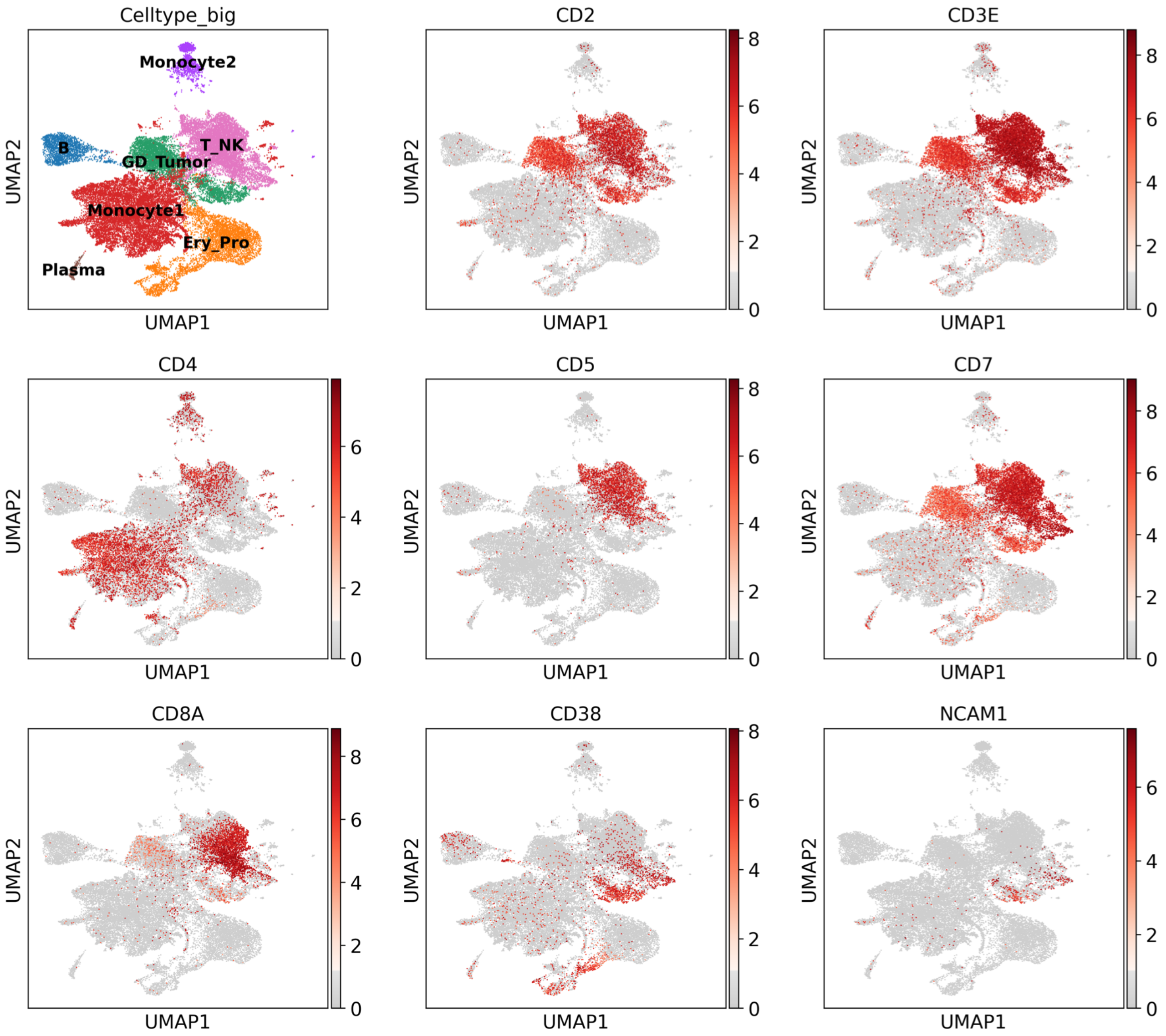

Figure S4. Sub-clusters identified in Ery_Pro. (A) UMAP of sub-clusters in Ery_Pro. Gran: Granulocyte progenitors, Multi_Pro: multipotent progenitors (B) The expression of marker genes for to the sub-clusters annotation

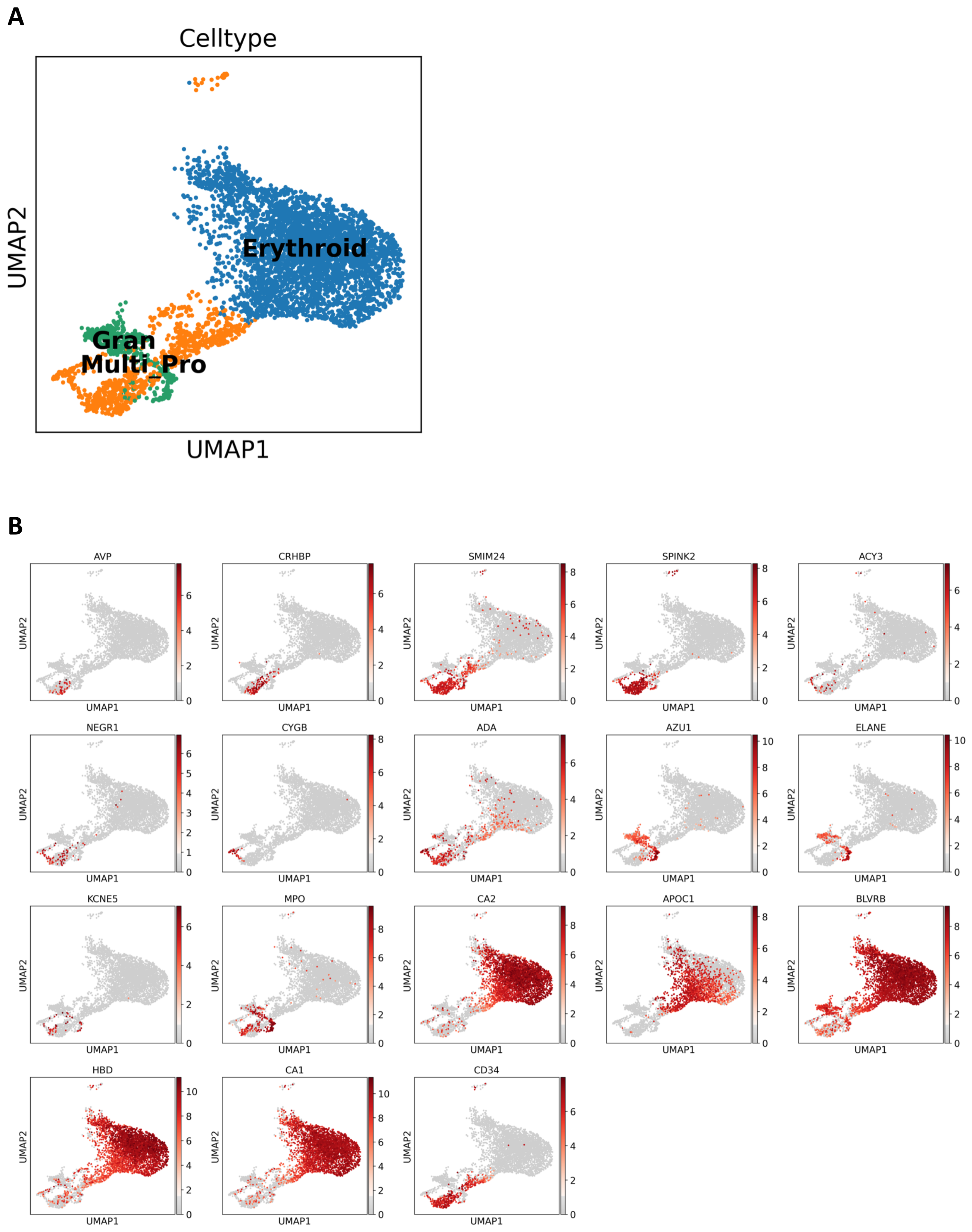

Figure S5. Cytotoxic genes reported to be expressed in normal γδ T cells had no or low expression in malignant γδ T cells

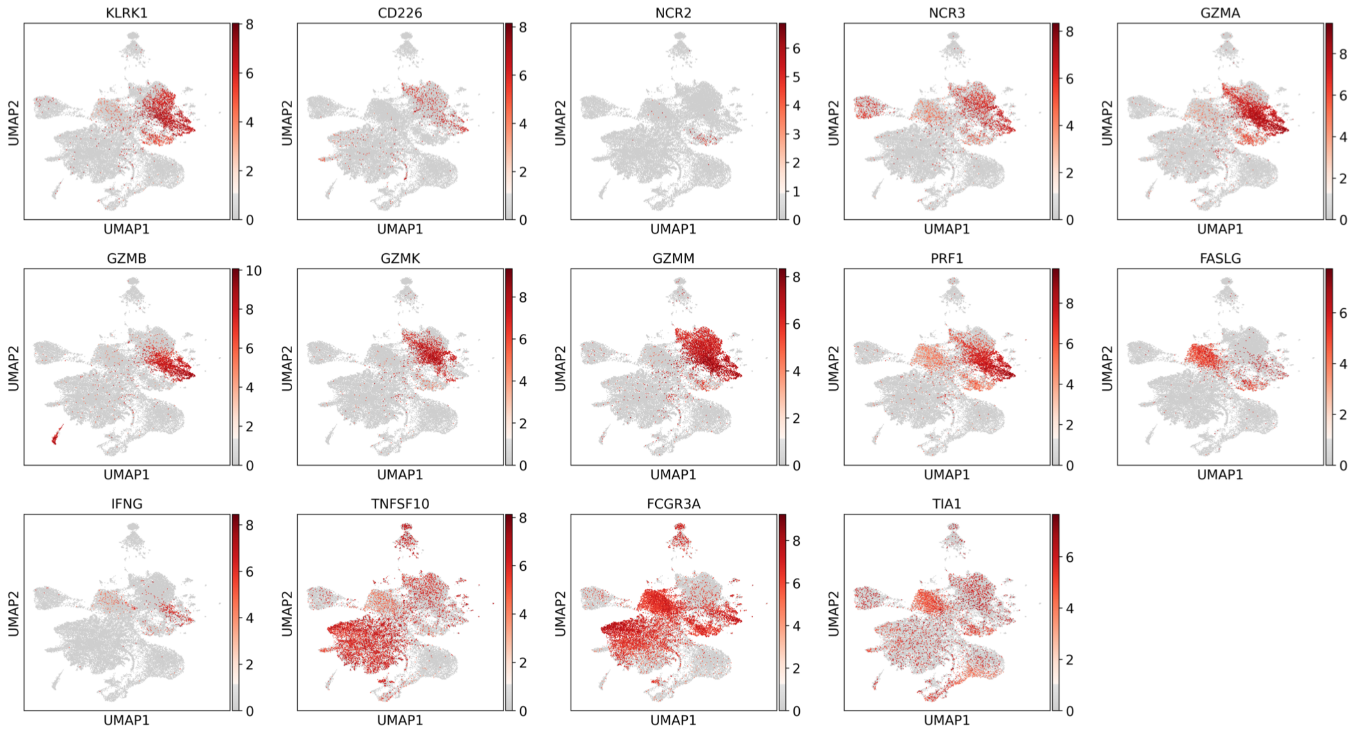

Figure S6. Cell Cycle phase distribution in Tumor sub-clusters

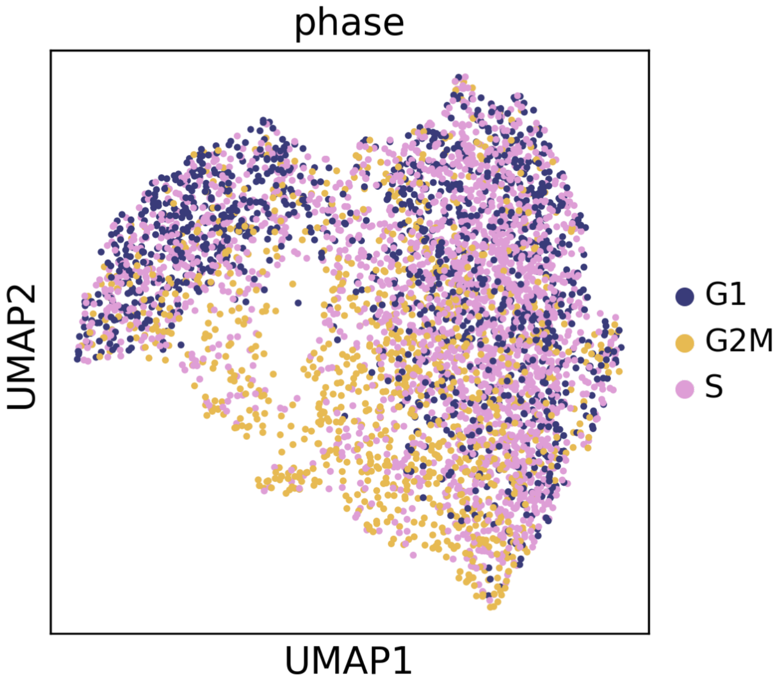

Figure S7. CD19 was highly expressed in Tumor_1 but not Tumor_2. Other B cell marker were not expressed in both Tumor sub-clusters

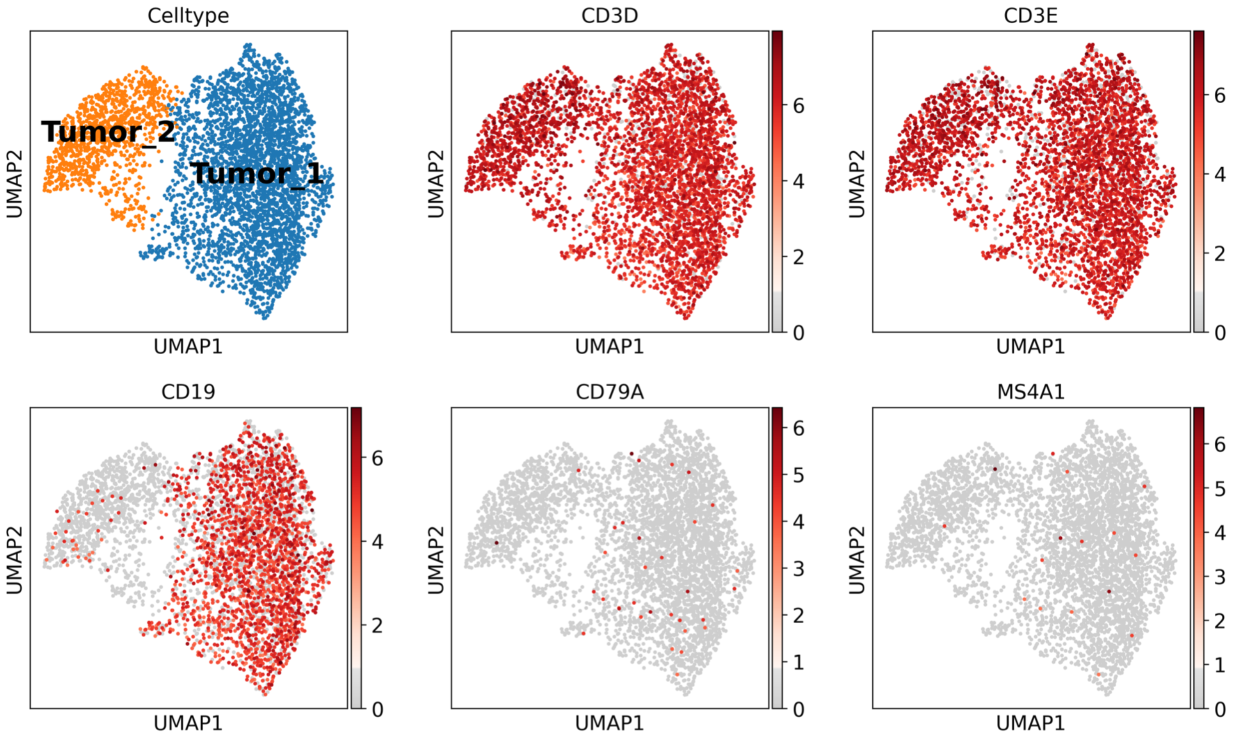

Figure S8. T cell sub-clusters identified from the tumor microenvironment. (A and B) The expression of marker genes for the T cell sub-clusters annotation

A

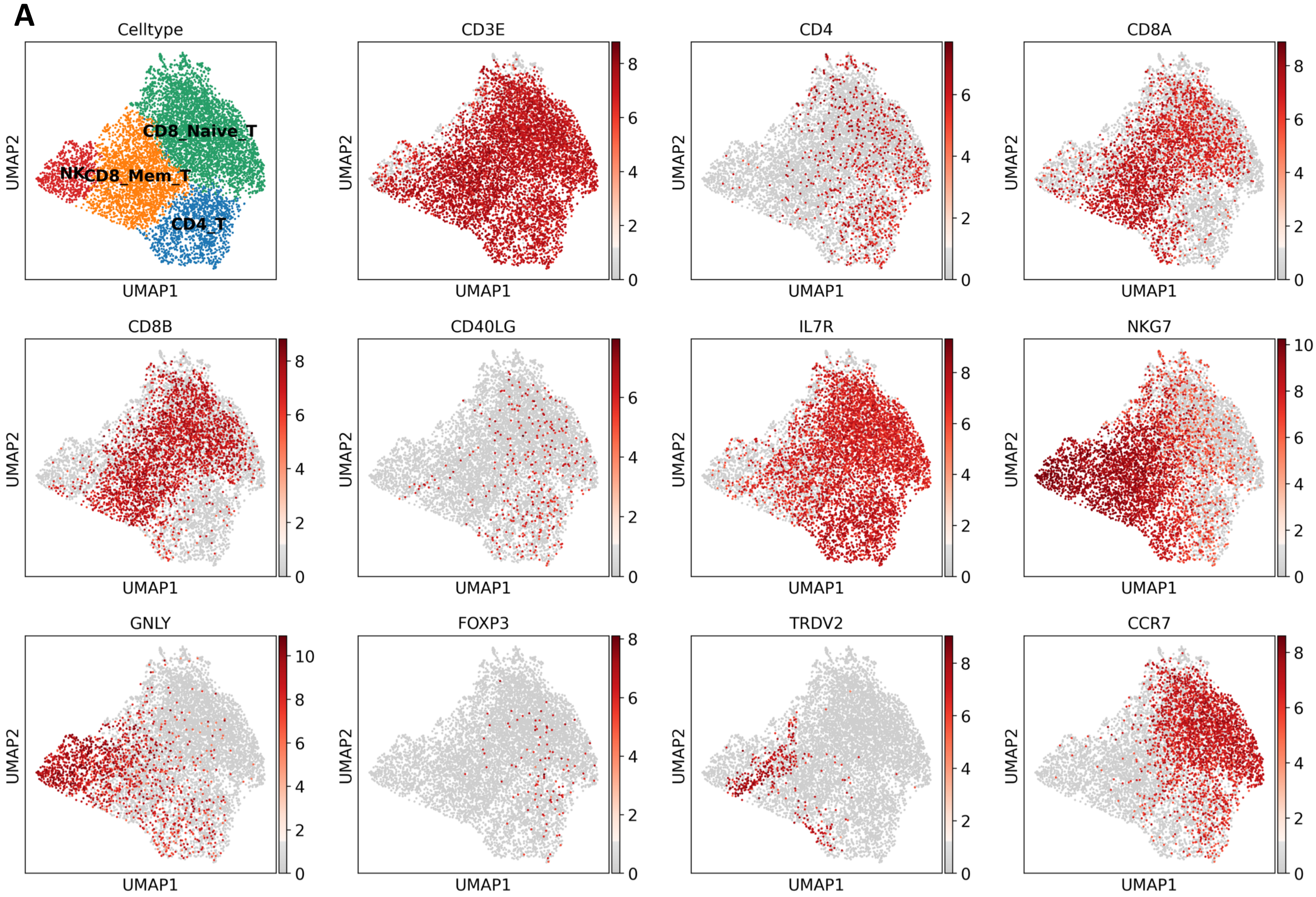

B

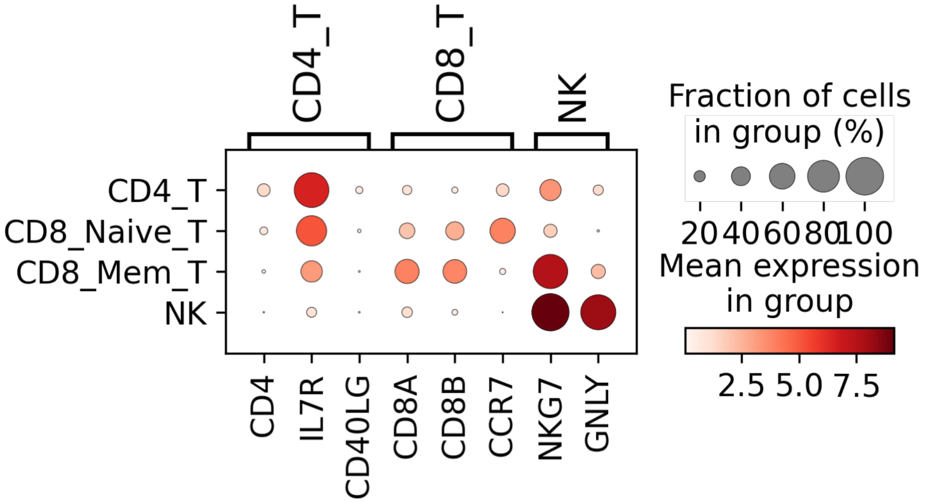

Figure S9. Total interaction counts and interaction strength inferred from each sample

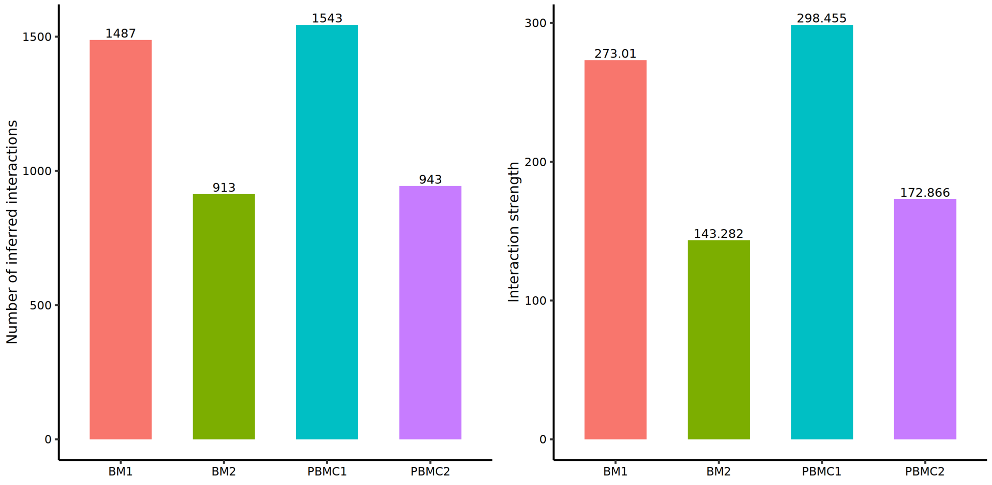

Supplement: Supplementary file 2 — Supplementary file2 (PDF 10652 KB) [file 13402_2022_745_MOESM2_ESM.pdf]
